# Experimental and field comparisons of two common methods for measuring microzooplankton grazing rates

**DOI:** 10.3389/fmicb.2025.1706193

**Published:** 2025-12-05

**Authors:** Jennifer L. Beatty, Brittany P. Stewart, Kendra A. Turk-Kubo, Debbie Lindell, David A. Caron

**Affiliations:** 1 Department of Biological Sciences, University of Southern California, Los Angeles, CA, United States; 2 Department of Ocean Sciences, University of California, Santa Cruz, Santa Cruz, CA, United States; 3 Technion—Israel Institute of Technology, Technion City, Haifa, Israel

**Keywords:** mortality, grazing, microzooplankton, *Prochlorococcus*, picophytoplankton

## Abstract

**Introduction:**

Grazing on picoplankton by microbial eukaryotes is a fundamental process within aquatic food webs, particularly in oligotrophic regions that are typically dominated by picophytoplankton. Remarkably, classical methods that have been used for decades to measure this process in the field have rarely been evaluated under controlled laboratory conditions where true rates of prey mortality can be quantified and compared to experimental results. This study evaluated two commonly used field techniques to estimate phytoplankton mortality rates by microbial consumers, the dilution technique and the disappearance of fluorescently labeled bacteria (FLB).

**Methods:**

An elaborate laboratory experiment was first conducted comparing picophytoplankton mortality rates measured using these two techniques to rates observed directly in culture based on changes in prey abundance, using the cyanobacterium, *Prochlorococcus*, as prey for a nanozooplanktonic grazer, *Paraphysomonas bandaiensis*. Secondly, a field experiment was performed in the North Pacific Subtropical Gyre off Hawai’i to compare the mortality rates estimated by the two methods.

**Results:**

Summed across multiple treatments in the laboratory, mortality rates estimated by FLB disappearance displayed high variability and on average underestimated observed mortality rates by ∼27%. The dilution technique also underestimated observed mortality rates (by ∼54%) but displayed lower variance (yielding an approximately 27% difference between the two methods). In contrast to laboratory results, field experiments resulted in an order of magnitude difference between grazer-mediated mortality rates using the two methods.

**Discussion:**

Our laboratory results revealed that both methods yielded reasonable albeit somewhat underestimated mortality rates in the laboratory setup, while differences between the two methods in our field studies implied that the underlying assumptions of one or both methods were not met. These findings advocate for caution in interpreting quantitative assessments of protistan-based mortality rates using these long-used approaches.

## 1 Introduction

Grazing by microbial eukaryotes is a key trophic process in aquatic ecosystems. Consumption by phagotrophic protists facilitates the movement of carbon to higher trophic levels and also contributes to nutrient recycling (Sherr and Sherr, 2002; Worden et al., 2015). Grazing on picoplankton is a dominant trophic process in oligotrophic regions of the world’s oceans and many lakes where picophytoplankton and bacteria constitute most of the standing stock of biomass and are generally too small for effective capture by many mesozooplankton. The heterotrophic protistan taxa conducting this vital trophic process display a vast array of feeding strategies across a wide spectrum of taxonomic groups (Jürgens and Massana, 2008; Caron et al., 2012). Additionally, diverse mixotrophic protists (Mitra et al., 2016; Stoecker et al., 2017) contribute to both rates of primary production as well as grazing, complicating their contribution to microbial trophodynamics. All microbial eukaryotic grazers demonstrate prey selectivity relating to prey size, shape, chemistry, motility, and perhaps other characteristics (Sherr and Sherr, 2002; Jürgens and Massana, 2008). These features of protistan grazing present significant challenges when measuring rates of prey consumption or prey mortality. Developing a method to measure grazing that does not disrupt the complex trophic interactions at play, or choosing a surrogate prey that accurately mimics rates of consumption by microbial eukaryotic grazers, has been a long and non-trivial endeavor.

Two commonly employed approaches developed to quantify trophic interactions between microbial consumers and picoplanktonic prey are the dilution technique, and the use of fluorescently labeled bacteria (FLB) as surrogate prey (Landry and Hassett, 1982; Sherr et al., 1987; Landry et al., 1995; Caron, 2000). These methods, and variations of them, are by far the most extensively employed approaches for estimating microbial grazing rates in pelagic marine and freshwater ecosystems.

The dilution technique to measure phytoplankton growth and mortality (grazing) rates relies on serially diluting the microbial grazers in a water sample and measuring the response of phytoplankton growth rates following incubation (usually 24 h) of the dilution series (Landry and Hassett, 1982; Landry et al., 1995). Nutrients are typically added to all bottles in the series prior to incubation in order to ensure that phytoplankton growth is the same at all dilutions levels (to compensate for nutrients produced by grazers, which have been serially removed), and unenriched, unfiltered water is also incubated to establish net phytoplankton growth rates in the presence of grazers without nutrient enrichment (i.e., “natural” phytoplankton growth and mortality rates) (Landry and Hassett, 1982; Landry et al., 1995). Apparent phytoplankton growth rates observed at each dilution of the series are plotted against the degree of dilution, and the slope of the line is calculated to determine the phytoplankton mortality rate attributable to grazing. There are a few basic assumptions inherent in this method: phytoplankton intrinsic growth rate is exponential and unchanged across the dilution series, while per-cell clearance rates of consumers are maximal and unchanged across the dilution series (although the rates of prey encounter and consumption are affected by dilution).

The uptake or disappearance of FLB (Sherr et al., 1987; Jürgens and Massana, 2008) have been employed in a wide range of aquatic systems to estimate grazing on picoplankton including heterotrophic bacteria and picophytoplankton (Jürgens and Massana, 2008; Weisse et al., 2016). For experiments examining community-level rates of prey mortality, FLB are added to water samples at tracer amounts (to avoid changing feeding rates due to altered prey abundances), incubated, and the “disappearance rate” of FLB is determined, usually using microscopy or flow cytometry. The main assumption of the method is that grazers consume and digest FLB (surrogate prey) during the incubation period at the same rates as natural prey. Filtered water samples (i.e., with grazers removed) containing FLB are also incubated to account for non-grazer-related changes in FLB abundances (e.g., adsorption to the walls of the container).

Both the dilution technique and FLB disappearance method have been employed for decades to measure microbial grazing rates across space and time (Calbet, 2008; Schmoker et al., 2013; Rocke et al., 2015; Medina et al., 2017), and the biogeochemical significance of the resulting estimates (Neuer and Franks, 1993; Calbet, 2008). Due to the pivotal nature of these estimates, several publications have explored the challenges associated with the assumptions of the methods. It has been suggested that the dilution technique overestimates grazing, that dilution can impact both grazer and prey populations, and that trophic cascades within dilutions may alter grazing rates during incubation (Dolan and McKeon, 2005; Agis et al., 2007; Calbet, 2008; Beckett and Weitz, 2017). Yet, other reports have tended to support the estimates provided by this method (Landry and Calbet, 2004; Saló et al., 2010; Simó et al., 2018). The FLB method is primarily susceptible to errors associated with the choice of the surrogate (e.g., size, taste, etc. can impact the grazing rate measurements), and the model chosen for calculating grazing rates from the rate of FLB disappearance from a water sample. The strengths and weaknesses of the use of FLB for estimating mortality rates of bacteria-sized particles have been detailed several times (Florenza and Bertilsson, 2023).

Despite these confounding issues, and the widespread use of both techniques up to the present time, relatively few studies have attempted to quantify these potential errors. The accuracy of these methods for determining mortality rates in field studies is difficult to ascertain because there is no objective mechanism for testing whether the assumptions of either method have been fulfilled. A major objective of the present study was therefore specifically to test the accuracy of the methods within a carefully controlled laboratory experiment in which the mortality rates were also determined directly from changes in prey abundance. In this way, the accuracy of both methods was tested against reality (i.e., mortality determined from actual changes in prey abundance). A second goal was to directly compare the results of the two methods to one another since both methods are rarely carried out simultaneously.

The experimental system focused on the mortality rate of a single picophytoplankton, *Prochlorococcus*, mediated by the heterotrophic nanoplanktonic protist (*Paraphysomonas bandaiensis*) that readily consumed the cyanobacterium. The study was conducted as a minor component of a larger experimental setup (Lindell et al., in prep) to compare protistan and viral mortality of the cyanobacterium, and therefore included treatments containing the protist and a virus that infects *Prochlorococcus.* The latter treatments allowed an additional analysis of the potential impact of viral mortality on the accuracy of the grazing rate estimations. Subsequently, the dilution technique and the FLB disappearance method were employed in field experiments to estimate picophytoplankton grazer-mediated mortality rates at two depths in the euphotic zone of the oligotrophic North Pacific Subtropical Gyre (NPSG). Taken together, our findings indicate that the dilution and FLB disappearance methods have the ability to provide reasonable, albeit low estimates of phytoplankton mortality under “ideal” laboratory conditions. However, the methodological assumptions of one or both methods were not met in our field applications, yielding significantly different estimates of grazing mortality. Caution should be exercised when interpreting the results of either method as quantitative assessments of microbial trophic interactions, and the performance of multiple grazing experiments whenever possible may improve the overall accuracy of rate estimates of this vital ecological process.

## 2 Materials and methods

Experiments comparing morality rates estimated using the dilution technique and the disappearance of fluorescently labeled bacteria were conducted in a large laboratory experiment and subsequently in a field setting. The overall approach and goals of the study are described in Table 1.

**TABLE 1:** Experimental approaches used and goals of each experiment.

Experiment setting	Techniques used	Goals
Lab experiment	FLB (FCM) DLN (FCM)	Compare the mortality rates determined by the FLB and DLN techniques to changes in prey abundances in culture, and to mortality rates estimated by both methods to each other
Field experiment	FLB (FCM) DLN (Chla)	Compare the mortality rates determined by the FLB and DLN techniques applied in field experiments to each other

The setting of each experiment, the techniques used [fluorescently labeled bacteria disappearance (FLB) or dilution technique (DLN)] and the method of analysis; flow cytometry (FCM) or fluorescence of total extracted chlorophyll *a* (Chla), and the goals of each experimental setting.

### 2.1 Laboratory-based experimental setup

**FIGURE 1:**
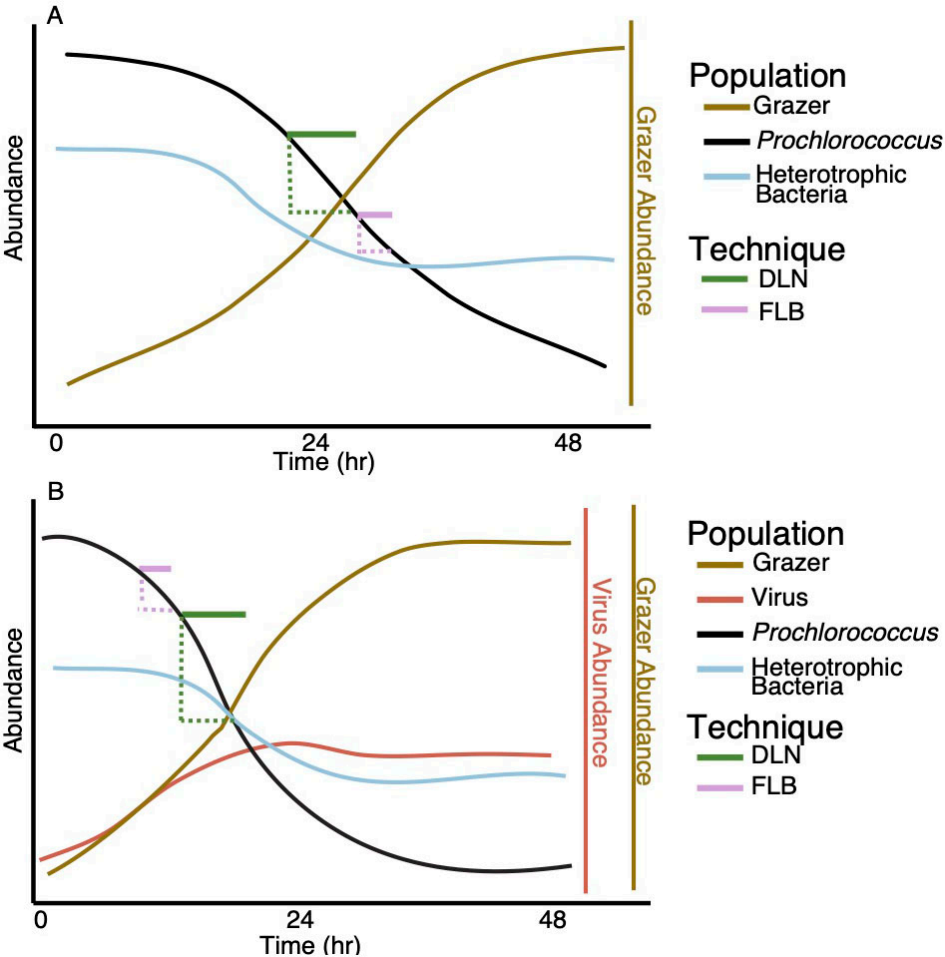
Idealized population changes through time in the laboratory experiment. Treatments with **(A)** *Prochlorococcus* in the presence of grazer only (i.e. no viruses), and **(B)** *Prochlorococcus* in the presence of grazer and virus. Horizontal solid purple lines indicate the time span of the dilution experiments (DLN), horizontal solid green lines indicate the time spans of the FLB disappearance experiments (FLB). The dashed colored lines indicate the time (horizontal) and abundance (vertical) ranges over which *Prochlorococcus* mortality rates in the cultures were determined and compared to dilution and FLB mortality rates over the corresponding time period.

The format of the experimental approach used in this study is described in detail in Lindell et al. (in prep). The overall goal of that investigation was to compare mortality of an important oceanic picophytoplanktonic cyanobacterium (*Prochlorococcus*) subjected to mortality mediated by protistan grazers and viral lysis, separately and in combination. The larger project was designed to yield insight into the ecology and biogeochemistry of oceanic ecosystems where *Prochlorococcus* dominates the phytoplankton community. The focus of our component of the larger study, presented here, provided an opportunity to compare the accuracy of two methodologies commonly used in field studies to estimate rates of microbial mortality mediated by protistan grazers within the carefully controlled laboratory experiment. The fundamental approach was to experimentally estimate mortality rates using the dilution technique and the disappearance of FLB, and compare those rates to the actual (i.e., observed) mortality rate of *Prochlorococcus* over the same time interval based on changes in cell abundances of the picophytoplankter in the cultures (shown conceptually in Figure 1 for one treatment, although multiple treatments were conducted).

The experimental design consisted of a picophytoplankter, a nanozooplanktonic grazer capable of consuming the picophytoplankter, and a cyanophage capable of lysing the picophytoplankter (Figure 1; Supplementary Figure 1). Experimental treatments were conducted with and without virus to examine protist-mediated mortality in the presence and absence of viral lysis of the phytoplankter. Grazers and viruses were inoculated into treatments at two levels (Supplementary Figure 1), although all treatments with virus were combined for analysis in this study since grazer-mediated mortality dominated mortality in all treatments. Treatments relevant to this study are conceptualized in Supplementary Figure 1 and included a control vessel containing *Prochlorococcus* alone (5 vessels), *Prochlorococcus* + high initial concentration of the grazer (3 vessels), *Prochlorococcus* + low initial concentration of the grazer (3 vessels), *Prochlorococcus* + low initial concentration of the grazer with virus (9 vessels), or *Prochlorococcus* + high concentration of the grazer with virus (9 vessels). Initial abundances in all treatments are provided in Supplementary Table 1 and were much higher than abundances observed in the natural environment, but were necessary to follow population dynamics on reasonable time scales. The data in Supplementary Table 1 represents a portion of the data presented in Lindell et al. (in prep) which provided a more in-depth analysis of grazer, virus and *Prochlorococcus* dynamics and biogeochemical transformations.

Briefly, experimental vessels of media in 10 L polycarbonate bottles were incubated for 48-h periods (which encompassed approximately three orders of magnitude decreases in *Prochlorococcus* abundance in treatments with protists). All bottles were kept in a walk-in incubator at 21°C on a 14:10 h light:dark cycle at 50 μmol photon m^–2^ s^–1^. A control treatment (i.e., without grazer or virus) contained solely the picoplanktonic cyanobacterium *Prochlorococcus* (*Prochlorococcus* sp. strain MED4). The experimental treatments relevant to this study contained either *Prochlorococcus* inoculated with a protistan grazer capable of consuming *Prochlorococcus* (*Paraphysomonas bandaiensis*, hereto referred to as the “grazer”), or the grazer together with a virus capable of lysing *Prochlorococcus* (P-SSP7, a T7-like cyanopodovirus that infects *Prochlorococcus* MED4 (Sullivan et al., 2003); hereto referred to as the “virus”). Both the *Prochlorococcus* and grazer cultures had mixed co-occurring heterotrophic bacterial assemblages present. The attendant bacterial flora grew in response to increases in organic material in the cultures as *Prochlorococcus* was consumed or lysed, presumably providing an additional nutritional source for the grazer (see Results and Discussion).

Samples were collected every 2 h for the measurement of abundances of *Prochlorococcus* throughout the approximately 48-h experimental periods. As depicted conceptually in Figure 1, this period of time represents the time between inoculation of the *Prochlorococcus* cultures with grazer or virus and rapid decline in *Prochlorococcus* abundances. All three measurements of *Prochlorococcus* mortality were made and compared multiple times during the 48-h period (dilution, FLB, and observed mortality by changes in cell numbers). Population abundances were analyzed on an Influx flow cytometer fitted with 457 and 488 nm lasers and a small particle detector (BD Biosciences, San Jose, CA) (Lindell unpubl. data). Samples were preserved at a final concentration of 0.1% glutaraldehyde for 15 min in the dark, flash frozen in liquid nitrogen and stored at −80°C until analyzed. *Prochlorococcus* was detected from forward scatter and red fluorescence (692 ± 20 nm).

### 2.2 *Prochlorococcus* mortality rates estimated using the dilution technique

Dilution experiments (Landry and Hassett, 1982; Landry et al., 1995) were conducted to estimate *Prochlorococcus* mortality due to grazing using water from each treatment bottle (therefore, at least in triplicate) during the 48-h experiment, during the time frame of rapid decrease in the abundances of *Prochlorococcus*. All dilution experiments were performed in small volumes due to multiple analyses being conducted within the larger experimental context (Lindell et al., in prep), and for much shorter periods of time than traditional dilution experiments because the elevated abundances of predators and prey resulted in rapid changes in abundances that allowed for short incubations. A subsample from each bottle was collected in a 500 mL flask, and a dilution series was prepared using 0.1 μm filtered fresh medium due to volume constraints from the treatment bottle. Each dilution series was performed in triplicate and consisted of 100% undiluted culture (150 mL culture), 20% diluted (120 mL culture, 30 mL fresh media), 60% diluted (60 mL culture, 90 mL fresh media), and 80% diluted (30 mL culture, 120 mL fresh media). Additional nutrients were deemed unnecessary because they were already present in excess in the culture medium. Following dilution series preparation, initial samples of 1.5 mL were collected and preserved with 10% formalin (made from 37% formaldehyde, MilliporeSigma, United States) at a final concentration of 1% formalin and stored at −80 °C until analysis by flow cytometry. Dilution flasks were incubated in the walk-in incubator for the duration of the incubation (4–12 h, depending on the treatment). Final samples of 1.5 mL were collected from each flask and preserved as for initial samples. All samples (initial and final) were analyzed on an Influx flow cytometer equipped with a 488 nm laser at the University of California, Santa Cruz (BD Biosciences, San Jose, CA). *Prochlorococcus* abundances were detected and quantified using forward scatter and red fluorescence (692 ± 40 nm).

Phytoplankton mortality rates were calculated using Model I linear regression of apparent growth rate versus dilution factor. The slope of the regression of apparent growth rate versus dilution factor yielded the mortality rate (m, units: d^–1^) (Landry and Hassett, 1982). Rates that were undetectable from zero were treated as “0” for subsequent analysis. In total, after outliers were removed (described below), 4 dilution experiments were conducted in the “grazer only” cultures and 23 in the “grazer and virus” cultures. The difference in the number of experiments without or with virus owed to the greater number of the latter treatments in the larger experimental context (Lindell et al., in prep). In addition to the four-point regressions, two-point mortality rates were also calculated using only the 80 and 60% dilution bottles to examine whether the high initial abundances of prey may have violated the assumption of the method that the grazers exhibited maximal clearance rates.

### 2.3 *Prochlorococcus* mortality rates estimated from FLB disappearance

FLB disappearance experiments (Sherr et al., 1987; Jürgens and Massana, 2008) were also used to estimate rates of *Prochlorococcus* mortality due to grazing by *P. bandaiensis*, assuming that FLB disappearance mimicked *Prochlorococcus* consumption. FLB were prepared from a culture of *Dokdonia donghaensis* following a standard protocol (Sherr et al., 1987; Caron, 2000) due to its ease of culture, similar size to *Prochlorococcus* and the unavailability of a *Prochlorococcus* culture. Briefly, *D. donghaensis*, was grown in ZoBell medium, rinsed and starved in filtered seawater for 2 days, stained, heat killed with 5-(4,6-dichlorotriazin-2-yl) aminofluorescein (DTAF), rinsed and centrifuged three times to remove excess stain, and then aliquoted and stored at −80 °C until used for experiments.

All FLB experiments were performed in triplicate in 500 mL culture flasks, similar to the dilution experiments. Subsamples from each 10 L culture vessel were added to 500 mL culture flasks for a total volume of 150 mL. FLB were added to flasks at approximately 20–30% of the abundances of *Prochlorococcus* (determined from flow cytometer counts 2 h prior to initiating the FLB experiments) to facilitate flow cytometric enumeration. Controls composed of FLB in 0.1 μm filtered fresh media were performed concurrently to characterize non-grazing losses. All flasks were placed in the incubator with the original experimental 10 L culture vessels for the duration of the incubations. Samples were removed immediately after addition of FLB (time zero) and again after 3–12 h of incubation (depending on the trajectory of changes in the population abundances in a treatment), preserved for flow cytometry with 10% formalin (made from 37% formaldehyde, MilliporeSigma, United States) at a final concentration of 1% formalin and kept at −80 °C until analyzed. Sample analyses were performed using an Influx flow cytometer at the University of California, Santa Cruz and identified using green (533 ± 30 nm) and yellow (585 ± 40 nm) fluorescence (BD Biosciences, San Jose, CA).

FLB disappearance rates (g, units: d^–1^), and by proxy, *Prochlorococcus* mortality rates, were determined using the following equation where F_0_ and F_*t*_ are the abundances of FLB at the beginning and end of the incubation, respectively, (t) is the length of incubation in days (Marrasé et al., 1992):


$$g=l\,n\left(\frac{F_{t}}{F_{0}}\right)*\left(-\frac{1}{t}\right)$$

Mortality rates were calculated from the change in FLB abundance in control bottles conducted at six time points to account for changes in FLB abundance due to non-grazing effects. The mean value of FLB loss in the control bottle was 1.35 d^–1^ (generally a small component of the measured grazing rates), which was subtracted from calculated mortality rates determined in the experimental bottles. Rates that were undetectable from zero were treated as “0” for subsequent analysis. In total after outliers were removed (described below), 17 FLB experiments were conducted in the “grazer only” cultures and 81 in the “grazer and virus” cultures due to the larger number of treatments with virus in the larger experimental context (Lindell et al., in prep).

### 2.4 Observed *Prochlorococcus* mortality rates based on changes in abundances

Mortality rates in the *Prochlorococcus* cultures were also determined in each experimental treatment containing grazers (with or without virus) based on changes in *Prochlorococcus* abundances over the same time interval for which mortality rates were measured using the dilution or the FLB disappearance techniques. *Prochlorococcus* abundances were determined by flow cytometry as described in section 2.1. These observed *Prochlorococcus* mortality rates were calculated using the following equation, where P_0_ is the initial *Prochlorococcus* abundance, P_*t*_ is abundance at the end of the incubation, and t is the length of incubation in days:


$$g=l\,n\left(\frac{P_{t}}{P_{0}}\right)*\left(-\frac{1}{t}\right)$$

Rates that were undetectable from zero were treated as “0” for subsequent analysis.

### 2.5 Field sample collection

Samples were collected on the Simons Collaboration on Ocean Processes and Ecology (SCOPE) PARAGON I cruise (July–August 2021), near Station ALOHA (Supplementary Table 2). Water samples were collected at two depths (25 and 125 m) on three dates (25 July, 29 July, 2 August). Physical/chemical features of the water column were captured using a Niskin^®^ rosette equipped with conductivity, temperature, depth (CTD), chlorophyll fluorescence and oxygen sensors (SeaBird, Bellevue, WA). Sampling depths represented the upper mixed layer of the euphotic zone and the approximate depth of the deep chlorophyll layer. Water from the Niskin^®^ bottles collected at the two sampling depths was transferred into clean (5% HCl-washed and rinsed with deionized water) 20 L carboys using silicone tubing to minimize turbulence that can damage delicate plankton. Estimations of microzooplankton mortality rates were conducted using both the dilution and FLB methods on all samples, as detailed below. Experiments were initiated at night and incubations were conducted for 24 h, with duplicate sets of samples incubated on an in situ array and in on-deck incubators.

### 2.6 Phytoplankton mortality rates in field samples estimated by the dilution technique

Seawater from each of the two sampled depths was filtered through pre-acid washed 0.2 μm inline AcroPak (#12686, Pall) filter cartridges (FSW) using silicone tubing. Whole (unfiltered) seawater (WSW) was added to 500 mL bottles previously rinsed with 5% HCl and deionized water. A dilution series was conducted in triplicate at concentration of 25, 50, 75 and 100% WSW. Nutrients were added to all treatments to account for reduced nutrient availability due to the exclusion of grazers (final incubation concentration- 0.1 μM FeCl_3_, 0.7 μM NaH_2_PO_4_ ⋅H_2_O, 1 μM NH_4_Cl, 10 μM NaNO_3_) (Landry et al., 1995; Connell et al., 2018). Six replicate samples of 250 mL WSW were collected from the water used to set up experiments and filtered onto 25 mm diameter GF/F filters (nominal pore size 0.7 μm, #1825-025, Whatman) for chlorophyll analysis. Filters were placed in 2 mL cryovials and stored at −80 °C until analyzed. One set of dilution bottles was placed on an *in situ* array and incubated at the depths of collection, while another set was placed in on-deck incubators because previous work has indicated that *Prochlorococcus* growth can be adversely affected in on-deck incubators (Chisholm, personal communication). The on-deck incubations were carried out in an incubator at ambient surface water temperature and light attenuation according to each depth (25 m or 125 m). The in situ array was retrieved after 24 h and samples from the bottles processed. Bottles from the on-deck incubators were also processed after 24 h.

Chlorophyll samples (duplicates from each bottle) were thawed in the lab and extracted in 4 mL of 100% acetone in the dark for 24 h at −20°C. Samples were warmed for 30 min in the dark before analysis on a Trilogy Laboratory Fluorometer (#7200-000, Turner Designs, San Jose, CA) using the non-acidification method (Welschmeyer, 2003). Apparent growth rates based on changes in chlorophyll concentration, and mortality rates calculated using Model I linear regression of apparent growth rate versus dilution factor, were determined as detailed above for the laboratory experiment (section 2.2).

### 2.7 Picoplankton mortality rates in field samples estimated by FLB

FLB disappearance experiments were used to quantify protistan mortality of bacterial-sized particles in aliquots of the same water samples employed in the dilution experiments. Seawater from each depth was aliquoted into triplicate 500 mL bottles. FLB were added to bottles at 5 × 10^4^ FLB cells mL^–1^ (approximately ≤ 10% of natural bacterial abundance). Controls (FLB at the same abundance in 0.2 μm filtered seawater) were also performed in triplicate to account for non-grazing impacts on FLB abundance. Initial samples were preserved for flow cytometry with 10% formalin at a final concentration of 1% formalin and kept at −80 °C until analysis. Experimental bottles were incubated on the same in situ array used for the dilution experiments and incubated at the depth of sample collection. Duplicate sets of bottles were placed in the on-deck incubators used for the dilution samples. Samples from the in situ array and on-deck experiments were collected after 24 h, and all samples were processed by flow cytometry at the University of Southern California using an Accuri C6 plus (BD Biosciences, San Jose, CA). FLB were detected using green (533 ± 30 nm) and yellow (585 ± 40 nm) fluorescence. Mortality rates for bacterial-sized particles were determined using the equation described previously. The mortality rates calculated from the controls were subtracted from the experimental bottle calculated rates. Rates that were undetectable from zero were treated as “0” for subsequent analysis.

Data visualization and statistical analyses were completed in R 2024.12.1 (R Core Team, 2022). Kruskal-Wallis rank sum tests were conducted in the R “stats” package to test for significant effect of experimental method and virus presence on the percent differences. For comparison between mean percent difference and mortality experiment type and between the mortality rates estimated in the field by mortality type, Wilcoxon tests were conducted. Welch’s *t*-test was used to test the significance of the linear slopes.

## 3 Results

### 3.1 *Prochlorococcus* abundance dynamics in the lab experiment

**FIGURE 2:**
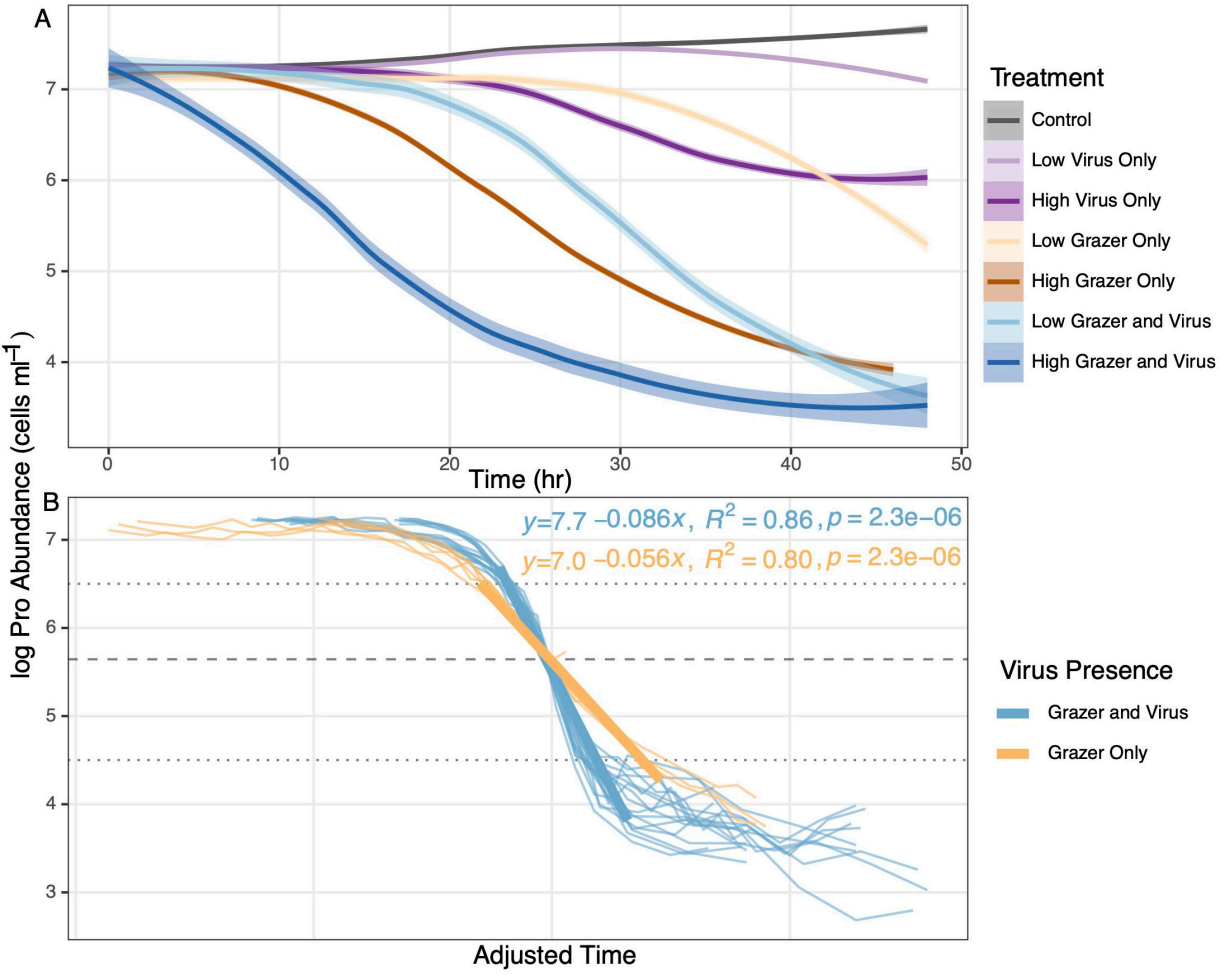
Changes in abundances of *Prochlorococcus* in all treatments. **(A)** Temporal changes (log base 10) of *Prochlorococcus* abundances grouped using loess regression with shaded areas denoting standard error. A portion of the data being presented is reproduced from Lindell et al. in prep. **(B)** Same data as in **(A)**, with time adjusted along the x-axis so that all curves converge at *Prochlorococcus* abundance of log 5.6 (i.e., approximately midway through the period of rapid prey decline in each treatment). Linear regressions (bold lines) are superimposed on a dataset for *Prochlorococcus* abundances > log 4.5 and < log 6.5. One treatment was excluded due to anomalous behavior. The slopes of the lines are significantly different from each other (Welch’s *t*-test *p* < 0.01). Colors in **(A)** indicate treatments with *Prochlorococcus* only control, high initial grazer abundances only, high initial grazer abundances with virus, low initial grazer abundances only, low initial grazer abundances with virus, high initial virus abundances only, and low initial virus abundances only. Colors in **(B)** distinguish treatments containing grazers only (yellow), and those with grazers and virus (blue) (i.e., treatments with low and high initial grazer abundances have been grouped).

Grazer-mediated mortality reduced *Prochlorococcus* abundances by 2–3 orders of magnitude generally within 48 h across all experimental treatments containing grazers (Figure 2A). Treatments containing only virus (i.e., no grazer) reduced *Prochlorococcus* abundances generally later and to a lesser degree compared to treatments with grazers present (Figure 2A). Unsurprisingly, treatments with initially high grazer abundances (with or without virus) reduced *Prochlorococcus* abundances more rapidly (generally within 24 h) than treatments initiated with low grazer abundances (with or without virus) (Figure 2A). This result coincides with earlier increases in the abundances of the grazer, *Paraphysomonas bandaiensis*, in treatments inoculated with higher abundances of the grazer (Supplementary Figure 2). Therefore, experiments were performed on short time scales (generally a few to several hours) relative to incubation times employed in most field experiments. Regardless of the incubation period, mortality rates estimated by the dilution technique or FLB disappearance method were compared to observed mortality rates determined from changes in prey abundance over the same time intervals.

The shapes of all *Prochlorococcus* abundance curves subjected to protistan grazing were generally consistent within each treatment (standard error shading in Figure 2A). However, exponential decreases in abundances occurred at different times during the 48-h experimental period due to minor variances in start times and/or initial predator/prey abundances. The dynamics of *Prochlorococcus* abundances in all treatments were therefore compared by superimposing the curves along the x-axis according to the point at which each treatment attained a *Prochlorococcus* abundance of log 5.6 (Figure 2B). The times along the x-axis were shifted between −20 and +6 h. This inflection point was chosen because it was approximately halfway between the initial and final abundances of *Prochlorococcus* (i.e., linear on a log-plot of abundance vs. time; Figure 2B). Superimposing the *Prochlorococcus* abundance curves addressed temporal offsets in the onset of rapid decreases in *Prochlorococcus* abundances between treatments yet allowed comparison of the shape of the abundance-time curves. The superimposed curves revealed that all the treatments demonstrated very similar dynamics for changes in *Prochlorococcus* abundances for treatments containing only grazers (Grazer Only) or grazers and viruses (Grazer and Virus) (yellow and blue lines in Figure 2B, respectively).

The comparison of mortality curves (Figure 2B) demonstrated only minor fluctuations in *Prochlorococcus* abundances from the start of each experiment until approximately log 6.5, when all treatments underwent rapid decreases in abundances. *Prochlorococcus* abundances also demonstrated divergence among treatments below log 4.5, presumably due to varying contributions of co-occurring bacteria to the nutrition of the grazer in the later stages of the experimental period (see Discussion). Therefore, the linear slope of each mortality curve was calculated for *Prochlorococcus* abundances between log 4.5–6.5 (Figure 2B). The slopes of the mortality curves in treatments containing the grazer and virus were highly consistent, as were curves in treatments containing only grazers, with the grazer and virus treatments exhibiting slightly more rapid decreases in *Prochlorococcus* abundances, as demonstrated by the steeper slope when grazer and virus were present than treatments containing only grazers, as might be anticipated (slopes averaged for the two types of treatments (grazers only, grazers with viruses) were significantly different from each other (Welch’s *t*-test, *p* < 0.01).

### 3.2 Observed vs. experimentally estimated mortality rates in the laboratory experiment

Mortality rates are strongly affected by the abundances of *Prochlorococcus* prey and their predators, therefore rates were interpreted in relation to the log-averaged *Prochlorococcus* abundances during the time that each experiment was performed, calculated using the equation of Heinbokel (1978).


$$P=\frac{\left(P_{2}-P_{1}\right)}{l\,n\left(P_{2}\right)-l\,n\left(P_{1}\right)}$$

where $\bar{P}$ is the average abundance of *Prochlorococcus* during each experimental time interval, *P_1_* is the abundances of *Prochlorococcus* at the initial time point, and *P_2_* is the abundances of *Prochlorococcus* at the final time point. Emphasis was placed on measuring mortality rates at intermediate *Prochlorococcus* abundances during the experiments. At high prey abundances near the time of inoculation with grazers, the small abundances of grazers often resulted in non-significant changes in prey abundance over the incubation period for dilution or FLB experiments. At low prey concentrations toward the end of the 48-h experiments, heterotrophic bacterial growth stemming from nutrients released by grazing could lead to an additional prey source for grazers, thereby affecting mortality rates of *Prochlorococcus* estimated by the two methods.

**FIGURE 3:**
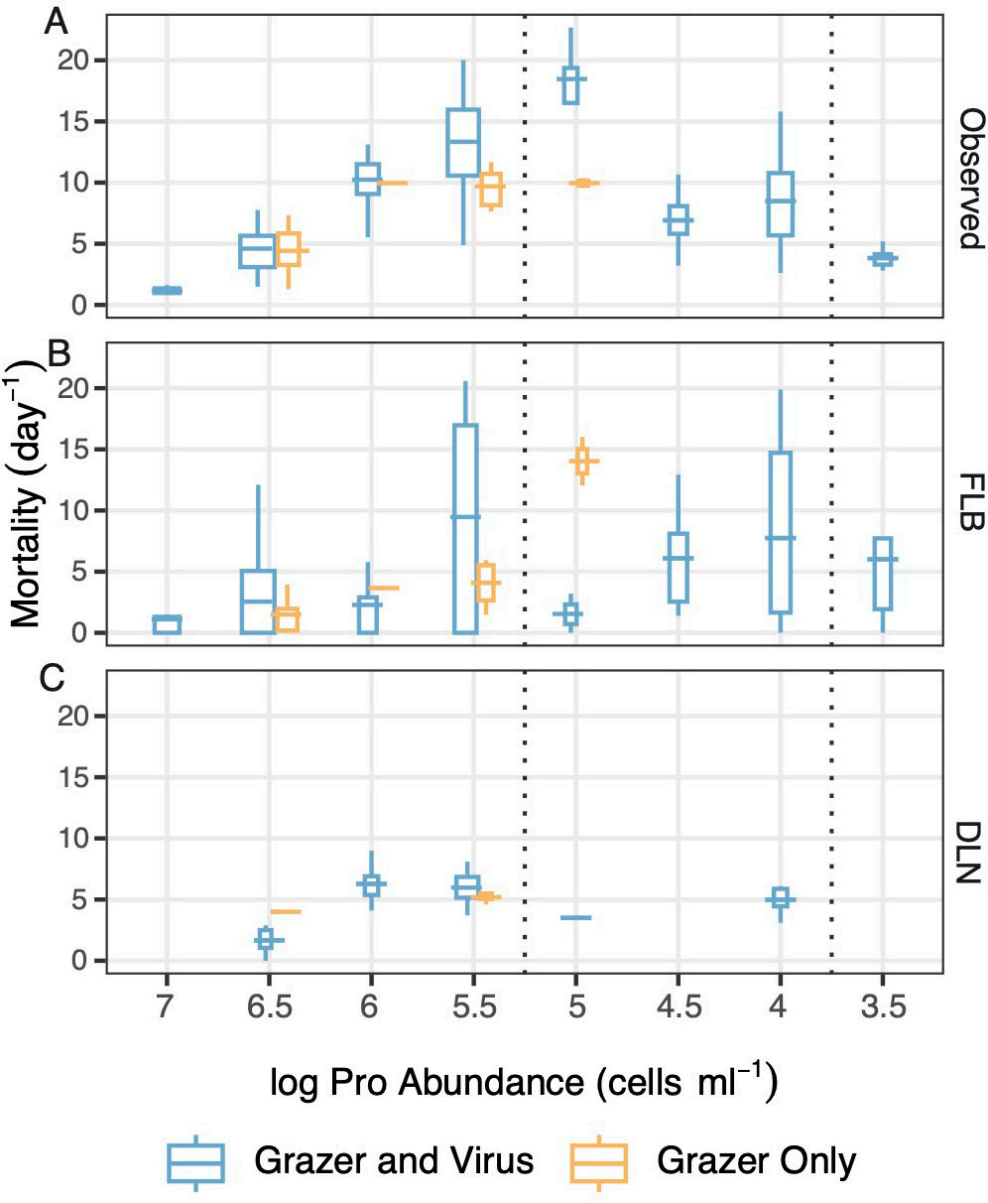
*Prochlorococcus* mortality rates across a range of *Prochlorococcus* abundances in the laboratory experiment including all treatments, as determined **(A)** from changes in *Prochlorococcus* abundances (Observed), **(B)** using the FLB disappearance method, and **(C)** using the dilution technique (DLN). The x axis is the binned log base 10 of average *Prochlorococcus* abundances for the time a FLB or dilution experiment was conducted. The x-axis has been reversed to reflect the changes in *Prochlorococcus* abundances through time from high to low abundances as the experiment progressed. Experiments yielding non-detectable rates were treated as zeros and outliers have been removed (see Methods and materials for details). Colors represent treatments with only grazers (yellow) or grazers and viruses (blue). For the boxplot, the upper and lower hinges represent the first and third quartiles, horizontal lines are means, and whiskers extend to 1.5× the upper or lower inter-quartile range (IQR).

*Prochlorococcus* mortality rates estimated or measured in three ways (experimentally estimated using the dilution technique, FLB disappearance, and rates determined directly from changes in *Prochlorococcus* abundances) were therefore compared across treatments at similar abundances of *Prochlorococcus* (assuming that prey abundance was a primary determinant of grazer-mediated mortality; Figure 3). Mortality rates determined across treatments, method and time intervals ranged widely, with overall averages of 5.14 ± 0.37 day^–1^ for the dilution experiments (*n* = 27), 4.24 ± 0.57 day^–1^ for the FLB experiments (*n* = 98), and 7.63 ± 0.45 day^–1^ when calculated directly from changes in *Prochlorococcus* abundances (*n* = 125; 27 conducted in conjunction with dilution experiments, 98 in conjunction with FLB experiments; Table 2). When mortality rates at similar average *Prochlorococcus* abundances were compared, there were relatively minor and inconsistent differences in mortality rates observed due to the presence or absence of virus (blue and yellow symbols in Figure 3, respectively), indicating the dominant role of grazer-mediated mortality in all treatments with grazers. Significant virus-mediated mortality would also have been revealed as consistently greater mortality rates in the grazer and virus treatments measured directly from decreases in *Prochlorococcus* abundances versus rates estimated using the FLB method (the latter method would assess losses from grazing but not viral lysis).

**TABLE 2:** *Prochlorococcus* mortality rates in the laboratory experiment, grouped by experimental technique and treatment.

Technique	Treatment	Experimentally estimated mortality (day^–1^)	Observed mortality (day^–1^)	Percent difference (%)
DLN	Grazer only (*n* = 4)	4.9 ± 0.4	7.4 ± 0.5	−34 ± 4
	Grazer and virus (*n* = 23)	5.2 ± 0.4	12.5 ± 0.7	−57 ± 4
FLB	Grazer only (*n* = 17)	3.9 ± 1.0	7.2 ± 0.8	−49 ± 12
	Grazer and virus (*n* = 81)	4.3 ± 0.7	6.3 ± 0.6	−22 ± 11

Values are mean and standard error of *Prochlorococcus* mortality rates (day^–1^) estimated using the dilution technique (DLN) or the disappearance of fluorescently labeled bacteria (FLB) experiments, for treatments containing only grazers or grazers with virus. Outliers have been removed.

Across the approximately 48-h experimental period, mortality rates of *Prochlorococcus* observed in the cultures based on changes in *Prochlorococcus* abundances displayed generally higher mortality rates at *Prochlorococcus* abundances between log 5 and 6, and lower rates at higher and lower *Prochlorococcus* abundances (Figure 3A). In contrast, mortality rates estimated using the dilution technique were generally low, and did not display any clear relationship to *Prochlorococcus* abundances (Figure 3C). Rates estimated using the FLB method (Figure 3B) were on average higher but more variable than the rates obtained using the dilution technique, and more consistent with rates observed directly from changes in *Prochlorococcus* abundances. The experiments were conducted throughout the day and therefore could have been affected by their performance in the light versus the dark. However, there was no apparent relationship between mortality rates obtained from any of the three methods and the light cycle at the time that each experiment was conducted (Supplementary Figure 3A).

### 3.3 Experimentally-determined mortality rates of *Prochlorococcus* differed from observed rates

**FIGURE 4:**
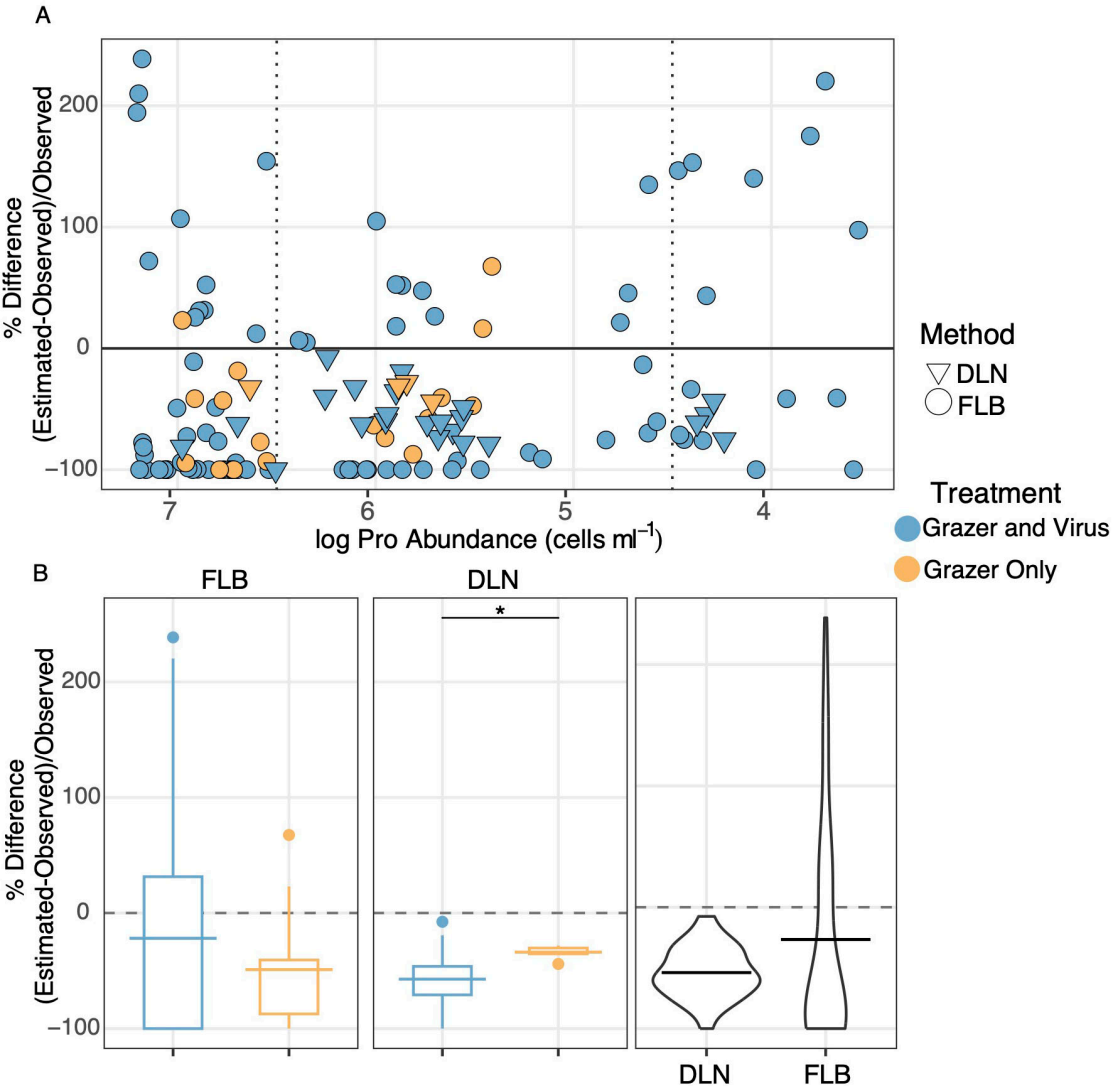
Percent differences in *Prochlorococcus* mortality rates in the laboratory experiment between experimental estimations by FLB disappearance (FLB) or dilution technique (DLN), and direct measurements (based on changes in *Prochlorococcus* abundances). Outliers were calculated separately for FLB and DLN based on the interquartile region. **(A)** Percent differences plotted against the averaged log abundance of *Prochlorococcus* at the type of each experiment. Colors are based on treatments with only grazers (yellow) or grazers and viruses (blue). Symbol shapes indicate FLB (circle) or DLN (triangle). **(B)** Boxplots where upper and lower hinges represent the first and third quartiles, horizontal lines are means, and whiskers extend to 1.5× the upper or lower inter-quartiles, horizontal lines are means, and whiskers to 1.5× the upper and lower inter-quartile range (IQR) (“*” indicates significant difference at *p* < 0.05, Wilcoxon test with p adjusted). The black violin plots include all data for the given experimental method, FLB or DLN, including both the treatments with only grazers, and with grazers and viruses. Experiments yielding non-detectable rates were treated as zeros and outliers have been removed (see Methods and materials for details).

Percent differences between the two methods for estimating *Prochlorococcus* mortality rates (FLB disappearance and dilution technique) and the observed mortality rates (based on changes in *Prochlorococcus* abundances) were examined to explore the accuracy of the two estimation methods (Figure 4A). Outliers were identified for the FLB method or dilution technique using the interquartile region of percent difference values (Supplementary Figure 4); the values identified as outliers were removed from further analysis (3 values were removed from the dilution dataset and 11 values were removed from the FLB dataset, and their removal is taken into consideration in the number of samples listed in previous sections). The percent differences in rates of mortality (FLB versus observed, or dilution versus observed) were then plotted against the log of average *Prochlorococcus* abundances at the time of each experiment to investigate the dependence of mortality rate on *Prochlorococcus* abundance (Figure 4A).

There was considerable variability in the differences between estimated and observed mortality rates across the range of *Prochlorococcus* abundances at which experiments were conducted, with no obvious trend between treatments with or without virus present (blue and yellow symbols in Figure 4, respectively). Average mortality rates estimated using the FLB technique for treatments with and without virus were 27 ± 10% of the average observed mortality rates determined by changes in prey abundance with no clear difference with respect to the presence or absence of viruses (Figure 4B). On average, mortality rates estimated using the dilution technique for treatments with and without virus were 54 ± 4% of the average observed mortality rates (Figure 4B). The dilution technique underestimated mortality rates more when grazers and viruses were present (57 ± 4%) than when the grazers were the only source of the mortality (34 ± 4%; *p* < 0.01, Wilcoxon test with p-adjusted). This difference could be due in part to the small number of experiments that remained after the removal of major outliers from the grazer only treatments. The potential for high prey abundance to interfere with the accuracy of the dilution technique was examined by recalculating mortality rates based only on the highest dilutions in the dilution series. However, there was no significant difference in the accuracy of the method when using a truncated dilution series including only the 80 and 60% dilutions (45 ± 7%) compared to the full dilution series (57 ± 4%; Supplementary Figure 5). There was also no obvious influence of the light cycle on these relationships (Supplementary Figure 3B).

### 3.4 Comparison of the dilution technique and FLB methods in field experiments

Water column temperature, salinity, chlorophyll patterns and microbial abundances observed near station ALOHA during the field experiments in 2021 were typical for the region and season, with a distinct chlorophyll maximum at approximately 125 m (Supplementary Table 2). *Prochlorococcus*, *Synechococcus* and heterotrophic bacteria were the dominant microbial biomass at 25 m while eukaryotic picophytoplankton biomass was more prevalent at 125 m.

**FIGURE 5:**
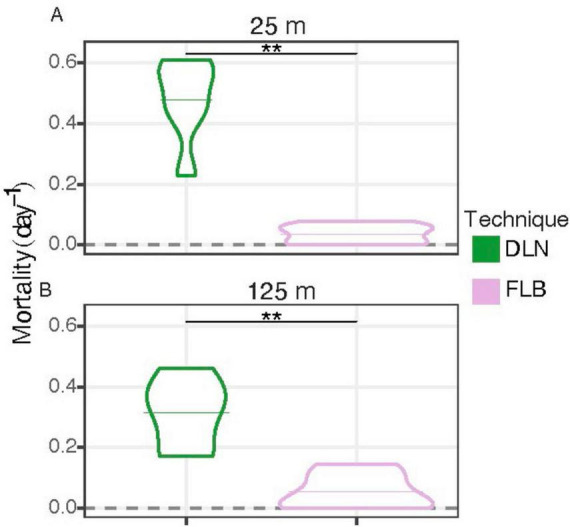
Violin plots based on phytoplankton mortality rates from field experiments, conducted with water collected at 25 m **(A)** and 125 m **(B)**. The dilution technique (DLN; green) was determined using total chlorophyll while FLB disappearance (FLB; purple) was determined using flow cytometry. Horizontal lines are means. Samples were combined by technique, depth, station, and incubation location. “**” indicates significant difference at *p* ≤ 0.01 (Wilcoxon test with Bonferroni adjusted *p*-value). Experiments yielding non-detectable rates were treated as zero.

Microbially-mediated grazing mortality rates measured using the dilution technique ranged from 0.17 to 0.61 d^–1^ with a mean of 0.40 d^–1^ (Table 3; Supplementary Table 3) for a total of twelve experiments conducted on three dates, at two depths, and incubated in situ or in on-deck incubators. All dilution experiments yielded detectable mortality rates. In contrast, grazing mortality rates measured using the FLB method ranged from “non-detects” (i.e., not significantly different from zero) to 0.10 d^–1^, with one outlier. Excluding the outlier, the average mortality rate estimated from the FLB method was 0.04 d^–1^ (including non-detects as zeros). Graphical comparison of mortality rates generated by the two methods from the same water samples and handled in the same manner indicated a significant difference of approximately one order of magnitude between the two methods, with the dilution method yielding significantly higher rates than the FLB method at both depths (Figure 5; **p < 0.01 Wilcoxon test with p adjusted).

**TABLE 3:** Mortality rates obtained in field experiments, grouped by experimental technique and depth of water collection.

Technique	Depth (m)	Mortality (day^–1^)
DLN	25 (*n* = 6)	0.48 ± 0.06
	125 (*n* = 6)	0.31 ± 0.05
FLB	25 (*n* = 6)	0.03 ± 0.01
	125 (*n* = 6)	0.05 ± 0.03

Values are mean and standard error of mortality rates (day^–1^) estimated using the dilution technique (DLN) using total chlorophyll a as a proxy for algal biomass, or the disappearance of fluorescently labeled bacteria (FLB). Outliers have been removed. Experiments yielding non-detectable rates were treated as zeros.

## 4 Discussion

The main objective of this study was to employ a carefully controlled laboratory experiment to assess the accuracy and precision of the dilution and FLB methods for measuring grazer-mediated mortality of a picoplanktonic prey. A secondary goal was to compare the dilution and FLB methods in side-by-side experiments conducted in the field. The laboratory experiment was conducted with high abundances of a single prey species (*Prochlorococcus*) to facilitate measurements in the lab and to shorten the duration of the experimental incubations for measuring mortality and thus enable multiple comparisons to be conducted. These abundances were not representative of natural conditions, but the trophic interactions investigated were representative of those occurring in natural water samples. An additional, minor goal of the study was to conduct treatments with grazers alone or grazers and viruses as a part of a larger study (Lindell et al., submitted) to consider possible impact of the presence of viruses on the grazer-focused methods examined here. Grazer-mediated mortality dominated *Prochlorococcus* mortality rates in all laboratory experimental treatments examined in this study. Viral lysis appeared to be additive to the impact of grazing, but always contributed a minor amount to *Prochlorococcus* mortality relative to grazing (Figure 2A).

Both dilution and FLB methods for measuring mortality rates underestimated the mortality rates determined directly from changes in prey abundance over the same time intervals examined for the two methods, although the variance among the dilution rates was less than variance among the FLB experiments (Figure 4). Averaged across all experiments, mortality rates determined by the FLB and dilution methods underestimated the observed rates of mortality by 27 and 54%, respectively (Figure 4). These findings indicate that under relatively ideal conditions (i.e., employing a carefully controlled and well-characterized culture system), the dilution and FLB approaches provided underestimates of *Prochlorococcus* mortality rates, with relatively high variability among individual experiments. One might therefore expect even lower accuracy and perhaps greater variability when attempting to quantify grazer-mediated mortality in a field setting where conformity to the assumptions inherent in these methods is unknown. Consistent with that expectation, we observed an order of magnitude difference between grazing rates estimated by these two methods when we compared the dilution and FLB approaches in the field (Figure 5), highlighting that caution should be taken when interpreting mortality rates derived from relatively few measurements.

### 4.1 Mortality-mediated changes in *Prochlorococcus* abundances in the laboratory experiment

Rates of decrease and the magnitudes of decrease in the abundances of *Prochlorococcus* during the laboratory experiment were substantially different when only viruses were present compared to treatments where grazers were present (either grazer only, or grazer and virus treatments; Figure 2A). Additionally, viruses did not reduce *Prochlorococcus* populations to the degree that grazers did, although these aspects of virus-mediated mortality would be influenced by the specific virus-prey pair (Fuhrman, 1999). In our experimental design, therefore, the virus was an additive but never dominant source of mortality for *Prochlorococcus* relative to the protistan grazer whenever grazers were present (compare rates of decrease of *Prochlorococcus* abundances in Figure 2A in the following treatments: “low virus only” versus “low grazer and low virus”; “high virus only” versus “high grazer and high virus”).

Rates of decrease in *Prochlorococcus* abundances were exponential (regression lines in Figure 2B in the presence of grazers alone or grazers and viruses), albeit with slightly different slopes owing to the contribution of virus-mediated mortality in the grazer and virus treatments (yellow and blue regressions in Figure 2B). The onset of substantive decreases in *Prochlorococcus* abundances was also temporally offset among the latter treatments primarily due to whether a treatment began with high or low initial concentrations of grazers. Slight differences in the initiation time of the treatments may also have contributed to these temporal offsets. Therefore, we normalized the curves to one another by overlaying them at a *Prochlorococcus* abundance of 5.5 (log 10). Additionally, decreases in *Prochlorococcus* abundance were observed earlier in the presence of viruses and grazers relative to grazers alone. The rapid decrease of *Prochlorococcus* abundance in the presence of grazers and viruses was expected given the multiple modes of mortality (grazer and virus) compared to a sole mode of mortality (grazer) (Simek et al., 2001; Berdjeb et al., 2011). Overlaying the mortality curves at the same log abundance of *Prochlorococcus* that was approximately ½ of the initial and final abundances observed in the treatments helped visualize differences in treatment effects (Figure 2B).

Comparison of the accuracy of the dilution and FLB methods (relative to observed mortality rates) was restricted to the range of *Prochlorococcus* abundances where direct measurements of mortality could be verified from significant changes in *Prochlorococcus* abundance. Grazer abundances at the early stages of the 48-h incubations were too low to significantly affect *Prochlorococcus* abundances over short experimental periods. Therefore, accurate measurements of changes in *Prochlorococcus* abundance by flow cytometry could not be obtained, and thus no comparisons of the two methods were performed during this early time period (i.e., at log *Prochlorococcus* abundances > 6.5 in Figure 2B). At the high initial *Prochlorococcus* abundances and lower grazer abundances, the grazers would likely be prey-saturated, invalidating one of the dilution technique’s fundamental assumptions of not altering the grazer clearance rate across the dilution series.

Conversely, the presence of a co-occurring bacterial assemblage in all treatments confounded a comparison of methods during the later portion of the incubations (i.e., at log *Prochlorococcus* abundances < 4.5 in Figure 2B). Bacterial abundances at the earlier stages of the 48-hour incubations were minimal (Lindell et al. in prep), and therefore should not have substantively affected rate measurements. However, as *Prochlorococcus* was consumed and organic material produced by the grazer and/or virus, that material undoubtedly stimulated cryptic growth of the heterotrophic bacterial assemblage during the latter part of the incubations. Consumption of co-occurring bacteria by the grazer (in addition to, or in lieu of the consumption of *Prochlorococcus*) would confound measurements of the mortality rate of *Prochlorococcus* by the grazer, at least for the FLB method which does not directly measure *Prochlorococcus* but uses FLB as a proxy for the consumption of all particles of appropriate size (both *Prochlorococcus* and heterotrophic bacteria at low *Prochlorococcus* abundances). For these reasons, dilution and FLB experiments were compared at log *Prochlorococcus* abundances > 4.5 and < 6.5.

In addition to the relative abundances of *Prochlorococcus* and the grazer, the light regime is another factor that might have affected a direct comparison of results from the dilution or FLB methods and the observed rates of mortality in our experimental setup. Our experimental incubations were generally short (a few hours) relative to the time employed in traditional dilution or FLB incubations carried out in the field (typically 24 h). Field measurements therefore generally span a full light cycle, whereas our grazing measurements were conducted completely during the dark cycle, completely during the light cycle, or a combination of both. We examined the potential impact of these different light regimes on the resulting accuracy and precision of the dilution and FLB estimations of mortality rates (Supplementary Figure 3). However, there were no discernible trends between observed mortality rates and the light regime, a finding consistent with at least one other study that found that light did not significantly impact grazing rates (Marrasé et al., 1992).

### 4.2 Limited accuracy of mortality rates by dilution and FLB methods

The potential caveats that might affect experimentally determined rates of *Prochlorococcus* mortality aside, rates estimated using the FLB method revealed a similar trend to the corresponding observed mortality rates, with highest values obtained at intermediate *Prochlorococcus* abundances (Figure 3). The general pattern of FLB estimated mortality rates mirrored the observed mortality rates, although underestimated the observed morality rates on average by 27% (Figure 4). Additionally, the FLB mortality rates exhibited high variability relative to the observed rates (Figure 3A vs. Figures 3B, 4).

The FLB technique relies on the uptake and digestion of FLB at the same rates that natural prey are ingested and digested. Differences in size, chemical composition, motility, live/dead and other factors would be expected to affect the ability of FLB to mimic prey ingestion either positively or negatively (Florenza and Bertilsson, 2023). Underestimation of mortality rates would occur if grazers selected against FLB in preference to living prey (Jürgens and Massana, 2008). Overestimation of mortality rates would occur if the predators preferentially consumed the surrogate prey. In our experiment, the size of the FLB was chosen to approximate the size of *Prochlorococcus*. We therefore speculate that our experimental design might yield superior accuracy to most FLB disappearance experiments conducted in the field because of our use of a prey of uniform size and composition (*Prochlorococcus*) and the choice of our FLB. Natural prey assemblages are composed of a mixture of prey sizes, shapes and species, and therefore might be less adequately represented by a single FLB type, as used in most FLB disappearance experiments. Some field studies have employed concentrated, labeled natural prey assemblages but the difficulty of obtaining such fluorescently labeled prey is significant, and their ability to replicate the natural assemblage is questionable (Cho et al., 2000; Cleven and Weisse, 2001; Pachiadaki et al., 2016).

Mortality rates estimated using the dilution technique displayed lower accuracy relative to observed mortality rates and relative to the FLB method (overall average 54% less than observed rates; 27% less than the FLB method) but yielded lower variability relative to the FLB method across a wide range of *Prochlorococcus* abundances (Figure 3C). In particular, the dilution technique failed to capture the very high mortality rates that were sometimes observed in the laboratory experiment based on changes in *Prochlorococcus* abundance (Figure 3A vs. Figure 3C). Because the dilution technique in our experimental design targeted *Prochlorococcus* specifically, similar mortality rates should have been observed if the assumptions of the method were met. Using fresh media diluted both the grazing pressure and viral interactions so both modes of mortality were reflected in the laboratory experiment.

A basic assumption of the dilution technique is that grazers are always exhibiting maximal clearance rates (i.e., dilution does not affect grazer feeding activity, only grazer abundance). Maximal clearance rate is only true if prey encounters are less frequent than the time required for prey to be ingested and processed. This is not likely the case at some of the highest *Prochlorococcus* abundances in the 48-h experiment, and the reason that we conducted the dilution experiments within a relatively narrow range of *Prochlorococcus* abundances. Further, the mortality rate estimations calculated using only the most diluted treatments (i.e., the truncated dilution series) were not significantly different from the mortality rates estimated using the entire dilution series (Supplementary Figure 5). This approach has been infrequently employed in an attempt to ensure that the clearance rates of grazers are always maximal. Our finding (no difference whether the entire dilution series was used or only a part of it) suggests that either the experimental set up did not invalidate the assumption of maximal clearance rates or that the assumption was invalidated even in the most diluted treatments, resulting in underestimation of the actual mortality rates. However, the importance of underestimating mortality rates at very high prey abundances, if it occurred, is questionable given that most field applications of the dilution technique have not involved highly elevated predator/prey abundances as employed in our laboratory experiment.

Another assumption of the dilution technique is that the growth rate of the prey (*Prochlorococcus*) is the same at all dilutions. This condition was ensured by supplementing all dilution bottles with nutrient rich medium, and thus seems unlikely as an explanation for underestimating mortality rates in the experiments.

### 4.3 Large variability in accuracy of techniques for assessing mortality rates

Both the dilution and FLB techniques yielded mortality estimates that often differed from observed mortality over the same incubation period (Figure 4). Variability was particularly high for rates obtained using the FLB method. These deviations from actual rates of mortality are concerning in part because such field applications are laborious to conduct and analyze (particularly true for the dilution technique), and as a consequence relatively few experiments are generally carried out because of time and labor constraints. The differences of the dilution or FLB results from observed mortality rates observed in our study call into question the accuracy of any particular experiment, and emphasize the importance of conducting multiple experiments to more accurately assess this important trophic process in nature.

Variances of the experimentally derived mortality rates (relative to observed rates) were not strongly affected by when each experiment was conducted within the 48-h experiment (shown when plotted against *Prochlorococcus* abundances; Figure 4A). Relatively poor matches were common between estimated mortality rates using the dilution or the FLB method relative to the observed rates for experiments conducted across > 3 orders of magnitude of *Prochlorococcus* abundance (Figure 4A), although the poorest matches were obtained at the lowest and highest *Prochlorococcus* abundances (for reasons noted in the previous section). These findings imply that there was inherent variability in the techniques not attributable to the predator and prey dynamics of individual experiments. Excluding the experiments conducted at very high and very low *Prochlorococcus* abundances (i.e., at the beginning and end of the 48-h experiment) removed a few experiments with large percent differences but did not change the overall trends (Figure 4B). High variability of experimentally derived mortality rates was observed even when the experiments were conducted during the optimal time period identified in Figure 2B (i.e., when grazer and *Prochlorococcus* interactions strongly dominated population dynamics within the experiment). Differences between observed and estimated mortality rates were also not attributable to the light conditions when the experiment was conducted, as noted above (Supplementary Figure 3B).

FLB disappearance appeared to more accurately estimate observed mortality rates than dilution on average, yet it yielded more variable results among the individual experiments (Figure 4). Although we observed less variability in treatments with grazers alone compared to the variability when grazers and virus were present (Figure 4B), there was no statistically significant difference between the means. If real, the higher variability when grazer and virus were present may reflect a difference in prey preference due to viral infection, however it is unclear if it would be a preference for or against the infected prey. Overestimation of mortality rates (i.e., values above 0% difference, Figure 4A) could have resulted from grazers consuming heterotrophic bacteria (and concurrently FLB), at a higher rate than grazers were consuming *Prochlorococcus*. Consuming more heterotrophic bacteria than *Prochlorococcus* would have been more likely at lower *Prochlorococcus* abundances toward the end of an experiment when cryptic bacterial growth was likely highest. Yet, overestimation of mortality using the FLB method also occurred at high *Prochlorococcus* abundances when heterotrophic bacterial abundance was relatively low (left side of Figure 4A). Underestimation of mortality rates could also be due to grazers selecting against FLB in preference to living prey (Jürgens and Massana, 2008), although we would have expected to see this consistently across all abundances of *Prochlorococcus*.

Relative to the large deviances from observed mortality rates using the FLB method, the dilution technique exhibited less variability among experiments, although the average degree of underestimation was greater than that obtained in the FLB experiments (Figure 4). Only a few experiments yielded dilution rates that were equivalent to or slightly greater than observed mortality rates (Figure 4A). These findings do not substantiate previous speculations that the dilution technique may consistently overestimate mortality rates (Dolan and McKeon, 2005) but do call into question the quantitative accuracy of the method. Mortality rates determined using the dilution technique in the treatments with grazers and viruses underestimated the observed rates to a somewhat greater extent than in the grazer alone treatments (Figure 4B). Further analysis is needed to determine if the difference between the observed mortality and estimated mortality in the treatments with grazers and viruses was due to the additive effect of viral lysis or to some synergistic effect when grazers and viruses were present (Beckett and Weitz, 2017; Staniewski and Short, 2018). A modified dilution technique has been employed to capture viral induced mortality at the same time as grazer induced mortality (Evans et al., 2003; Staniewski and Short, 2018). This modified dilution technique was not employed in this study due to the already-complex study design.

### 4.4 Plankton mortality rates in field experiments

Experiments in the field to directly compare mortality rates determined using the dilution and FLB methods were conducted on three dates and at two depths in the NPSG (Figure 5; Table 3). The resulting picoplankton mortality rates differed on average by approximately one order of magnitude, with rates determined by dilution (0.40 d^–1^) substantially higher than rates determined by FLB (0.04 d^–1^). The FLB and dilution methods yielded significantly different rates at both depths (Wilcoxon test with Bonferroni adjusted *p*-value at *p* ≤ 0.01). Only minor differences could be attributed to the mode of incubations (i.e., in situ array versus on-deck incubator). We attempted to normalize these comparisons to the degree possible by conducting them using the same water samples, modes of incubation, and incubation periods of 24 h for all experiments to avoid diel changes in microbial grazing activities (Ribalet et al., 2015; Rii et al., 2016; Connell et al., 2020). Nonetheless, on average, we observed ≈10-fold higher rates by dilution technique than FLB disappearance (Figure 5) compared to only a 27% difference between methods in our laboratory experiment (Figure 4B). Moreover, the average mortality rate determined using FLB in the laboratory experiment underestimated the observed rate by only 27%, whereas the dilution technique underestimated the observed rate by 54%.

Deciding which of the methods (if either) employed in the field yielded a more accurate appraisal of picoplankton mortality was obviously not possible because there is no mechanism for “objective” determinations of prey mortality rate in the field study, as was specifically designed into the laboratory study. Moreover, these two experimental methods are based on fundamentally different assumptions. The dilution technique assumes that phytoplankton growth rates and microzooplankton grazing rates are not impacted by dilution. However, studies have documented that dilution can impact both grazer and prey communities (Dolan et al., 2000; Agis et al., 2007). Trophic cascades can also impact the linearity of mortality rates established using the dilution technique (Calbet, 2008), nutrient replenishment can differentially affect the growth of phytoplankton species, and low mortality rates can be difficult to detect (Schmoker et al., 2013). Due to such considerations, at least one study has concluded that the dilution method may overestimate grazing (Dolan and McKeon, 2005).

Perhaps a more fundamental issue in our application of the dilution technique in the field is that we were relegated to measuring phytoplankton mortality rates from changes in total chlorophyll. *Prochlorococcus* is a dominant component of the phytoplankton of the NPSG (60–70% of total chlorophyll; R. Letelier, pers. comm.), with *Synechococcus* and picoplanktonic eukaryotic algae constituting much of the remaining phytoplankton. These assemblages appear to be adequately assessed by chlorophyll analyses but the influence of other phytoplankton species on mortality rate measurements is unknown. Nonetheless, the grazing mortality rates estimated by the dilution technique in our field experiments (Supplementary Table 3) are within the range of biomass-normalized production rates reported for *Prochlorococcus* (0.3–0.9 d^–1^), *Synechococcus* (0.3–0.8 d^–1^) and photosynthetic picoeukaryotes (0.2–0.6 d^–1^) in the NPSG (Rii et al., 2018), in rough agreement with the mortality rates observed in dilution experiments. This suggests that, on average, the dilution technique may have been providing reasonable estimations of grazer-mediated mortality in our field experiments.

The major assumption of FLB disappearance experiments is that the tracer is consumed and digested at the same rate as natural prey. In our field experiments we used the same bacterium employed in our laboratory experiment, but natural picoplankton assemblages consist of a huge diversity of species with many different sizes and shapes. Microzooplankton grazers primarily select prey based on size but can also distinguish based on motility and chemical composition and other factors (Florenza and Bertilsson, 2023). Thus it seems probable that the mortality rates obtained in our field studies in general provided a poor estimation of mortality of the natural picoplankton assemblages present in the NPSG, yielding substantial underestimation of actual mortality rates and explaining differences in the rates obtained using the two techniques. While some studies have concentrated the natural bacterial assemblage and used it as the tracer prey instead of a monoculture FLB (Cho et al., 2000; Cleven and Weisse, 2001; Pachiadaki et al., 2016), such approaches are labor intensive and often impractical because they require prior access to the microbial community at the sampling location, and do not necessarily capture a representative sample of the picoplankton, and were not attempted in this study.

The disparity obtained in the present study between the two most-commonly employed field methods for estimating phytoplankton mortality rates raises questions regarding recent global assessments of microzooplankton grazing impact in the ocean. If our results are generally applicable, then we would expect there has been a general underestimation in current global assessments of microzooplankton grazing. This would be remarkable given that recent estimates already indicate that microzooplankton may account for the removal of 60–75% of daily phytoplankton production (Landry and Calbet, 2004). Such an underestimation would mean that microzooplankton may be the primary source of mortality for a very large percentage for phytoplankton, and imply pivotal roles for these microbial consumers in elemental cycling and as prey supporting all higher trophic levels in planktonic ecosystems.

## 5 Conclusion

Quantifying grazer-mediated mortality of picoplanktonic prey is crucial to understanding and modeling the fate of carbon in the ocean. We tested two widely employed methods, the dilution technique and FLB disappearance, in a carefully controlled laboratory experiment to investigate the accuracy and precision of these methods in a simple microbial food web including a grazer, a phytoplankton prey and a virus. We later applied the two methods in side-by-side experiments conducted in the North Pacific Subtropical Gyre. Mortality was dominated by the grazer in our laboratory experimental setup, while viral lysis had a minor additive impact to that of grazing. Both methods generally underestimated mortality rates relative to the observed mortality rates, in the presence of grazers alone and in the presence of viruses and grazers (by 27% for the FLB technique, and 54% by the dilution technique). Mortality rates determined using the dilution technique yielded smaller variance than the FLB disappearance method, relative to true rates determined by changes in prey abundance. Differences between the mortality rates determined using the two methods in field experiments were substantially greater than differences observed in the lab (≈10-fold), with the dilution technique yielding significantly higher mortality rates unlike in the lab. Our results highlight that caution should be exercised when interpreting the validity of any single estimate of grazer-mediated mortality using either the dilution or FLB methods, although the performance of multiple experiments in a given locale or situation may provide a reasonable albeit somewhat low estimations of this fundamental process of aquatic food webs.

## Data Availability

The R scripts used to analyze data and produce figures are available on GitHub: https://github.com/Jennifer1Beatty/promo.
