# Supplementary material for: Experimental and field comparisons of two common methods for measuring microzooplankton grazing rates
Source: Front Microbiol. 2025 Dec 5;16:1706193. doi: 10.3389/fmicb.2025.1706193 (PMC12714900; doi:10.3389/fmicb.2025.1706193)
Supplement: Supplementary file 1 [file Supplementary_file_1.docx]

**Supplemental materials**

**Methods for initial laboratory set up abundances**

Samples were collected every two hours for the measurement of abundances of *Prochlorococcus* and the grazer, and every six hours for the measurement of abundances of heterotrophic bacteria throughout the 48-hour experimental periods. Samples were analyzed on an Influx flow cytometer fitted with 457 nm and 488 nm lasers and a small particle detector (BD Biosciences, San Jose, CA) (D. Lindell unpubl. data). Samples were preserved at a final concentration 0.1% glutaraldehyde for 15 minutes in the dark, flash frozen in liquid nitrogen and stored at -80°C until analyzed. Samples for measurements of grazer and heterotrophic bacterial abundances were stained at a final concentration with 1X SYBR Green I and incubated for 15 minutes in the dark. *Prochlorococcus* was detected from forward scatter and red fluorescence (692 ± 20 nm), while grazers were detected by forward scatter and green fluorescence (530 ± 20 nm). Heterotrophic bacteria were detected by forward scatter and green fluorescence (530 ± 40 nm). Samples analyzed for viral abundances were taken every six hours and quantified by qPCR for encapsulated virus DNA. Samples were subjected to DNase treatment immediately after collection to remove free virus DNA, and then flash frozen in liquid nitrogen and stored at -80°C (no fixative). Samples were thawed, diluted 100-fold and processed for qPCR quantification using SYBR Green I qPCR assays in a LightCycler 480 qPCR machine (#05015278001, Roche Diagnostics).

**Supplemental Tables**

**Supplemental Table 1.** Abundances of *Prochlorococcus*, grazers, viruses and heterotrophic bacteria in the laboratory experiment at the time of inoculation of the *Prochlorococcus* culture with viruses and/or grazers. The mean and standard errors are based on treatment type as described in the methods (Low Grazer Only n=3, Low Grazer and Virus n=9, High Grazer Only n=3, High Grazer and Virus n=9).

| **Treatment** | **Pro MED4 (cells ml^-1^)** | **Grazer (grazers ml^-1^)** | **Virus (virus ml^-1^)** | **Heterotrophic Bacteria**  **(bacteria ml^-1^)** |
| --- | --- | --- | --- | --- |
| Low Grazer Only  (n=3) | 1.77 x 10^7^ ± 0.10 | 6.23 x 10^2^ ± 0.60 | - | 1.18 x 10^6^ ± 0.50 |
| Low Grazer and Virus  (n=9) | 1.69 x 10^7^ ± 0.40 | 7.85 x 10^2^ ± 1.31 | 1.79 x 10^7^ ± 0.40 | 7.98 x 10^5^ ± 0.10 |
| High Grazer Only  (n=3) | 1.63 x 10^7^ ± 0.70 | 4.68 x 10^3^ ± 0.20 | - | 1.81 x 10^6^ ± 0.04 |
| High Grazer and Virus  (n=9) | 1.32 x 10^7^ ± 0.10 | 7.43 x 10^3^ ± 1.1 | 1.84 x 10^8^ ± 0.60 | 9.19 x 10^5^ ± 1.51 |

**Supplemental Table 2.** Environmental and biological conditions at each station where dilution technique and FLB disappearance experiments were performed in the NPSG. Chlorophyll and temperature were obtained from the CTD casts from which water was collected for experiments. Values for picoplankton abundances were obtained by flow cytometer as described by the HOT data repository ([https://hahana.soest.hawaii.edu/hot/protocols/protocols.html#](https://hahana.soest.hawaii.edu/hot/protocols/protocols.html)).

| **Station**  **(DD/MM/**  **YYYY)** | **Latitude** | **Longitude** | **Depth**  **(m)** | **Chlorophyll**  **(mg L^-1^)** | **Temperature**  **(˚C )** | ***Prochlorococcus***  **(cells mL^-1^)** | ***Synechococcus***  **(cells mL^-1^)** | **Pico-eukaryote**  **(cells mL^-1^)** | **Heterotrophic Bacteria**  **(cells mL^-1^)** |
| --- | --- | --- | --- | --- | --- | --- | --- | --- | --- |
| G1  25/07/2021 | 21.734 | 155.277 | 25 | 0.20 | 25.7 | 2.25 x 10^5^ | 6.29 x 10^2^ | 9. x 10^2^ | 6.29 x 10^5^ |
|  |  |  | 125 | 0.56 | 22.5 | 4.71 x 10^4^ | 4.09 x 10^1^ | 1.47 x 10^3^ | 2.96 x 10^5^ |
| G2  29/07/2021 | 21.567 | 156.003 | 25 | 0.12 | 25.8 | 2.19 x 10^5^ | 1.11 x 10^3^ | 1.11 x 10^3^ | 6.40 x 10^5^ |
|  |  |  | 125 | 0.46 | 22.3 | 3.61 x 10^4^ | 0 | 1.99 x 10^3^ | 3.07 x 10^5^ |
| G3  02/08/2021 | 22.135 | 156.262 | 25 | 0.13 | 25.7 | 2.52 x 10^5^ | 1.11 x 10^3^ | 8.07 x 10^2^ | 6.18 x 10^5^ |
|  |  |  | 125 | 0.74 | 22.4 | 9.53 x 10^4^ | 2.55 x 10^2^ | 1.78 x 10^3^ | 3.76 x 10^5^ |

**Supplemental Table 3.** Mortality rates obtained using the dilution technique (DLN) and FLB methods observed in field experiments near station ALOHA (A Long-term Oligotrophic Habitat Assessment) in the North Pacific Subtropical Gyre, by experiment and incubation location. ND indicates values undetectable from zero; treated as ‘0’ in Figure 5. Asterisk (*) indicates on outlier not included in Figure 5.

| **Station** | **Depth (m)** | **Incubation**  **Location** | **DLN Mortality Rates**  **(d^-1^)** | **FLB Mortality Rates**  **(d^-1^)** |
| --- | --- | --- | --- | --- |
| G1 | 25 | Array | 0.23 | 0.05 ± 0.02 |
|  |  | Deck | 0.58 | 0.08 ± 0.07 |
|  | 125 | Array | 0.34 | ND |
|  |  | Deck | 0.46 | ND |
| G2 | 25 | Array | 0.45 | ND |
|  |  | Deck | 0.61 | ND |
|  | 125 | Array | 0.17 | 5.86* |
|  |  | Deck | 0.4 | 0.14 ± 0.06 |
| G3 | 25 | Array | 0.45 | 0.02 ± 0.06 |
|  |  | Deck | 0.56 | 0.06 ± 0.1 |
|  | 125 | Array | 0.18 | 0.03 ± 0.03 |
|  |  | Deck | 0.33 | 0.10 ± 0.03 |

**Supplemental Figure Legends**

**Supplemental Figure 1.** Schematic of experimental treatments. Bottles represent each experimental treatment relevant to this paper, as described in Supplemental Table 1 and treatment colors are the same as in Figure 2. Text describes the average starting abundances of different cells in each treatment and the number of treatment bottles.

**Supplemental Figure 2.** Temporal changes in abundances of *Paraphysomonas bandaiensis* in all treatments containing the protist. Lines are based on loess and shaded areas are standard error. Colors in indicate treatments with high initial grazer abundances only, high initial grazer abundances with virus, low initial grazer abundances only, low initial grazer abundances with virus, high initial virus abundances only, and low initial virus abundances only. Colors are consistent with treatments in Figure 2A.

**Supplemental Figure 3**. *Prochlorococcus* mortality rates (A, as plotted in Figure 3) and percent differences between observed and experimentally determined rates in the laboratory experiment, plotted according to the light regime at the time of each experiment (B), with outliers removed. Colors reflect the experimental light conditions during each experiment. Dark: experiment was conducted entirely during the dark period; Dark-Light: experiment began during the dark period and finished in the light period; Light: experiment was conducted entirely during the light period; Light-Dark: experiment began in the light period and finished in the dark period. The upper and lower hinges represent the first and third quartiles, horizontal lines are means, and whiskers extend to 1.5 x the upper or lower inter-quartile range (IQR).

**Supplemental Figure 4.** Percent differences in *Prochlorococcus* mortality rates in the laboratory experiment between experimental estimations by FLB disappearance (FLB) or dilution technique (DLN), and direct measurements (based on changes in *Prochlorococcus* abundances) as plotted in Figure 4A, including outliers. Percent differences plotted against the averaged log abundance of *Prochlorococcus*. Colors are based on treatments with only grazers (yellow) or grazers and viruses (blue). Symbol shapes indicate FLB (circle) or DLN (triangle). The red dashed line represents the cutoff for outliers. Experiments yielding non-detectable rates were treated as zeros (see Methods and Materials for details).

**Supplemental Figure 5**. Percent differences between observed and experimentally determined mortality rates using the dilution technique in the laboratory experiment (as plotted in Figure 4), with outliers removed. Colors reflect whether the full dilution series (green) or only the 60% and 80% dilutions (orange) were used to calculate the mortality rate. (A) Percent differences plotted against the averaged log abundance of *Prochlorococcus*. Symbol shapes indicate full dilution series (circle) or the truncated dilution series (square) was used to calculate the mortality rate. (B) Boxplots where upper and lower hinges represent the first and third quartiles, horizontal lines are means, and whiskers extend to 1.5 x the upper or lower inter-quartile range (IQR).
