# Supplementary figures and images for: Experimental and field comparisons of two common methods for measuring microzooplankton grazing rates
Source: Front Microbiol. 2025 Dec 5;16:1706193. doi: 10.3389/fmicb.2025.1706193 (PMC12714900; doi:10.3389/fmicb.2025.1706193)

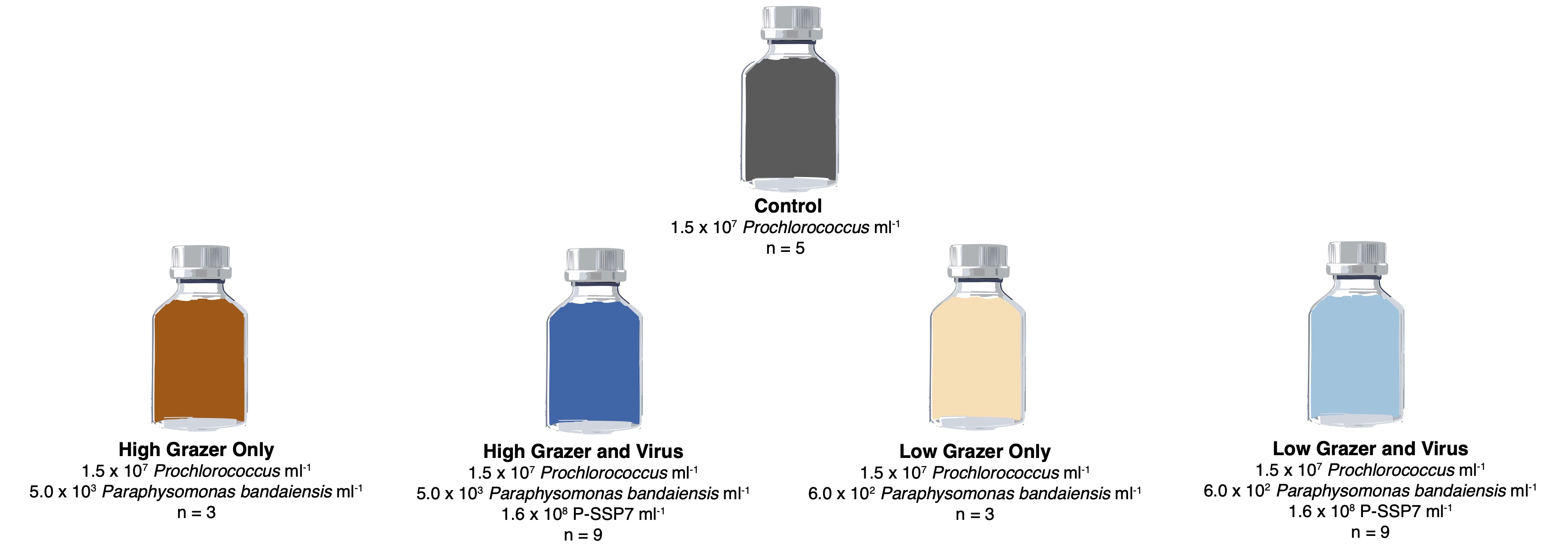

Supplement: Supplementary file 2 [file Image_1.jpeg]

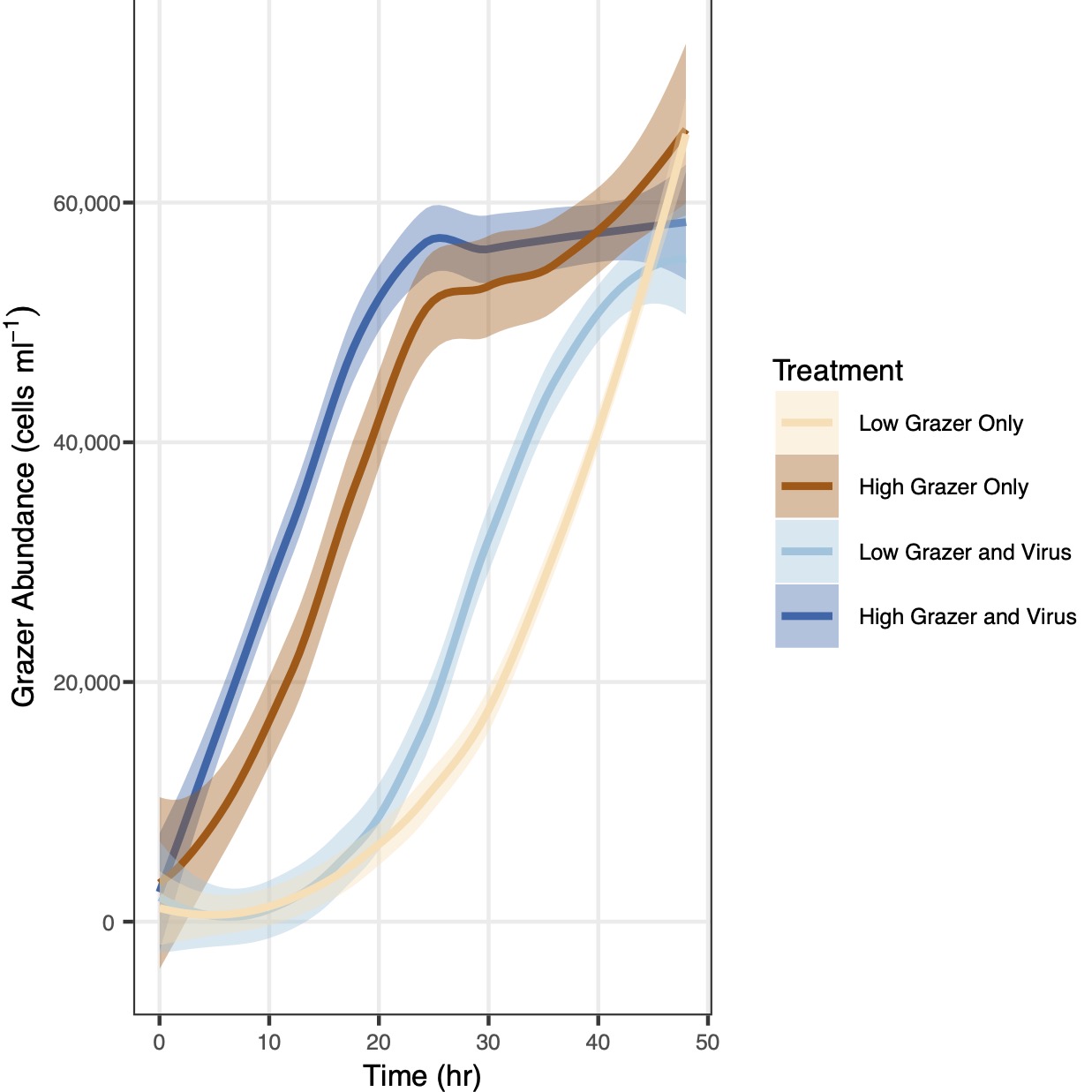

Supplement: Supplementary file 3 [file Image_2.jpeg]

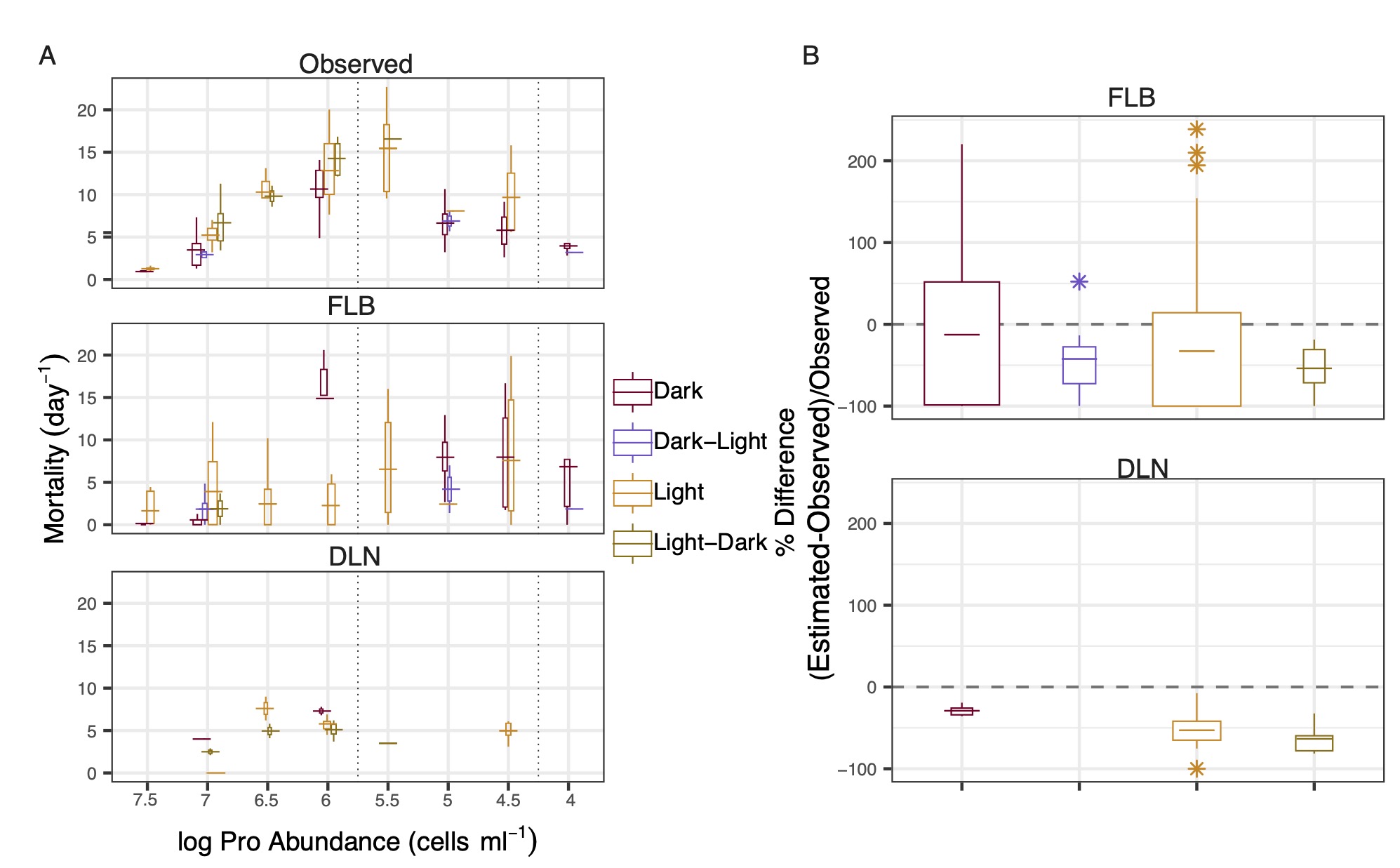

Supplement: Supplementary file 4 [file Image_3.jpeg]

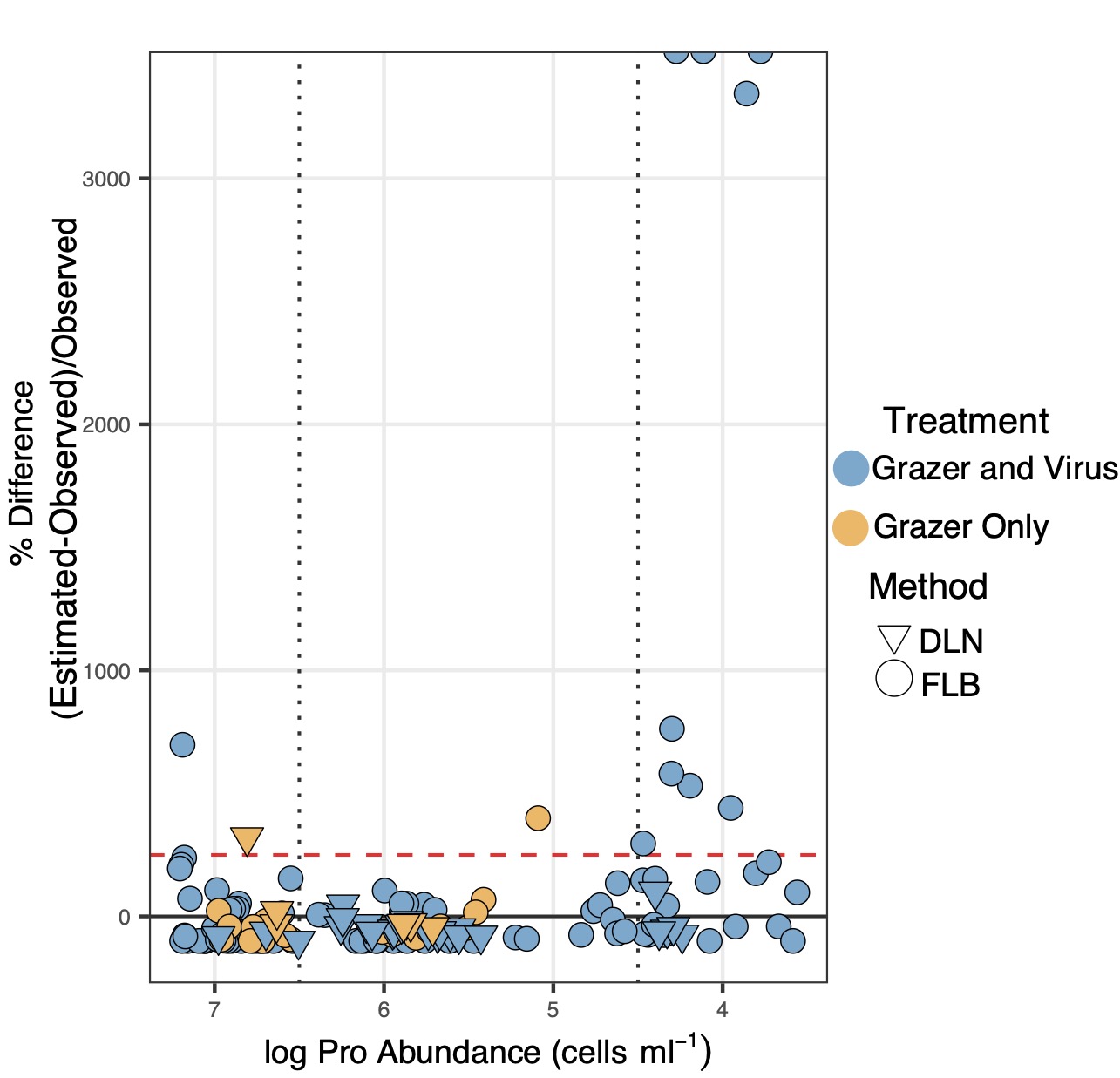

Supplement: Supplementary file 5 [file Image_4.jpeg]

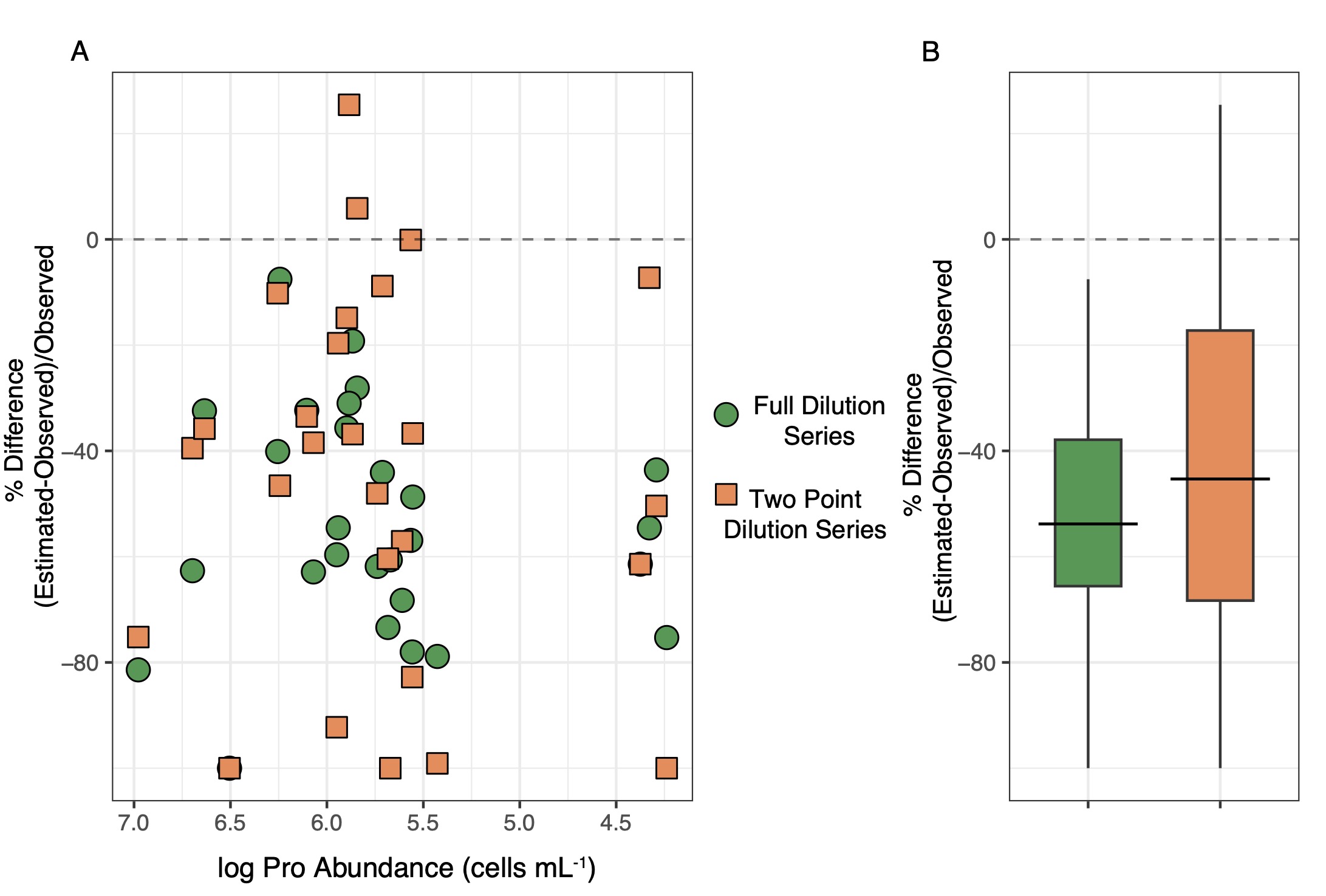

Supplement: Supplementary file 6 [file Image_5.jpeg]
